# Trajectories of learning approaches during a full medical curriculum: impact on clinical learning outcomes

**DOI:** 10.1186/s12909-021-02809-2

**Published:** 2021-07-07

**Authors:** Giovanni Piumatti, Sissel Guttormsen, Barbara Zurbuchen, Milena Abbiati, Margaret W. Gerbase, Anne Baroffio

**Affiliations:** 1 Faculty of Medicine, Unit of Development and Research in Medical Education (UDREM), University of Geneva, Geneva, Switzerland; 2 Division and Department of Primary Care Medicine, Geneva University Hospitals, Geneva, Switzerland; 3 Institute of Public Health, Faculty of BioMedical Sciences, Università della Svizzera Italiana, Lugano, Switzerland; 4 Faculty of Medicine, Institute for Medical Education (IML), University of Bern, Bern, Switzerland

**Keywords:** Approaches to learning, Student performance, Learning outcome, Growth curve modeling

## Abstract

**Background:**

No consensus exists on whether medical students develop towards more deep (DA) or surface learning approaches (SA) during medical training and how this impacts learning outcomes. We investigated whether subgroups with different trajectories of learning approaches in a medical students’ population show different long-term learning outcomes.

**Methods:**

Person-oriented growth curve analyses on a prospective cohort of 269 medical students (M_age_=21years, 59 % females) traced subgroups according to their longitudinal DA/SA profile across academic years 1, 2, 3 and 5. Post-hoc analyses tested differences in academic performance between subgroups throughout the 6-year curriculum until the national high-stakes licensing exam certifying the undergraduate medical training.

**Results:**

Two longitudinal trajectories emerged: *surface-oriented* (*n* = 157; 58 %), with higher and increasing levels of SA and lower and decreasing levels of DA; and *deep-oriented* (*n* = 112; 42 %), with lower and stable levels of SA and higher but slightly decreasing levels of DA. Post hoc analyses showed that from the beginning of clinical training, *deep-oriented* students diverged towards better learning outcomes in comparison with *surface-oriented* students.

**Conclusions:**

Medical students follow different trajectories of learning approaches during a 6-year medical curriculum. *Deep-oriented* students are likely to achieve better clinical learning outcomes than *surface-oriented* students.

**Supplementary Information:**

The online version contains supplementary material available at 10.1186/s12909-021-02809-2.

## Background

Seminal work by Marton and Säljö [1] and Biggs [2] described learning approaches as motivations and strategies students adopt to gain new knowledge, and distinguished between deep (DA) and surface learning approaches (SA). DA underlie intrinsic motivation and interest during learning, understanding the meaning of what is learnt, relating information to prior knowledge, looking for underlying principles and critically evaluating knowledge and conclusions drawn. SA relate to instrumental motivation for learning, reproducing content, memorising and rote learning in order to pass the tests. Learning approaches are central during higher education since they impact several learning outcomes [3–5]. In particular, they are determinant for academic achievement even after taking into account time spent in learning, gender and intellectual ability [3–7].

Training medical students to become competent doctors require them to develop, among others, competencies in reasoning skills, clinical problem solving and critical analysis [8], all skills strongly related to the use of DA. Indeed, learning approaches are significant predictors of academic performance for medical learners [9] and the amount of clinical knowledge acquired during clinical training as well as the success in final examination are positively associated with the use of DA during medical studies [10, 11]. Even more, the way students use learning approaches during medical school can predict their approaches to work when they will become practicing doctors [12]. It is thus crucial that medical schools create the conditions to promote and sustain deep learning during medical training. Therefore, pedagogical interventions need to be informed by empirical evidence from observational studies in order to maximise chances of producing tangible results. In addition, it remains unclear whether and how learning approaches change over time and impact students’ clinical learning outcomes.

How to encourage students to learn in-depth has been the subject of a myriad of studies and the emerging picture is contrasted and even contradictory [13, 14]. Explanations for these findings rely on the complexity of factors impacting learning approaches. On one hand, students can modulate their use of SA and DA depending on the educational context in which they are learning [14, 15]. However, the efforts put into promoting the use of DA through learner-centered teaching methods have been rather unsuccessful [14, 16]. Even worse, students seem more readily to shift from DA to SA, than the opposite [14, 16, 17]. On the other hand, specific student-related factors seem to influence how they use learning approaches and how they modulate their strategies in response to a given educational environment. Students might have personal predispositions towards using either SA or DA [18] and their initial use of learning approaches when entering university could be more predictive of the subsequent use of learning approaches than the educational context itself [19–21]. Moreover, some students might be stable, whereas others in the same learning context might change their use of learning approaches, revealing that even the most carefully designed learner-centered teaching environment may induce or prevent the use of DA [22]. Finally it appears that how students perceive their educational environment is even more influential than the context itself, thus questioning the efficiency of learner-centered environments [13, 23].

As it is essential to follow and support students’ strategies to good learning practices, authors recently called for a person-centered approach with a longitudinal perspective [20]. Indeed, major limitations of previous studies include measurements with only short-time intervals and analyses at the group rather than at the individual level [17]. To our best knowledge, no prospective long-term cohort study has evaluated how learning approaches evolve and influence the learning outcomes along an entire medical curriculum. Accordingly, the current study combined variable- and person-centered longitudinal analyses [24] to address two unanswered research questions: (1) Do subgroups with different trajectories of learning approaches exist in a given population of medical students? (2) If yes, do these subgroups have different learning outcomes?

Based on previous research, we hypothesised the presence of two groups of medical students with different trajectories of learning approaches and learning outcomes along the curriculum. More specifically, students more oriented in using DA (*deep-oriented* students) at the onset of their studies would keep or strengthen this predominant learning approach whereas students initially more oriented in using SA (*surface-oriented* students) would tend to reinforce their use of SA during medical studies. *Deep-oriented* students would then report better learning outcomes than *surface-oriented* students.

## Method

### Sample and procedures

 Participants were recruited among medical students enrolled during their first academic year at the University of Geneva, Switzerland, in 2011, 2012 and 2013. Data on learning approaches used in this study derived from students’ self-reported answers at an annual survey and student performance from examinations scores recorded by the Geneva Faculty of Medicine from Year 1 to 5 and by the Institute for Medical Education for the federal licensing exam (FLE) in Year 6. Participants provided their student ID in order to be matched throughout the duration of the study and to merge their self-reported answers with their examinations scores. Data were anonymised as student IDs were managed by a technical administrator. For the current analyses, we selected students that delivered at least three self-reports across four data collections: first/pre-selection study year (Year 1), beginning of pre-clinical training (Year 2), end of pre-clinical training (Year 3), and end of clinical training (Year 5). In total, 269 students (mean age at Year 1 = 20.85 years, SD = 1.92, range = 18–38, 59 % females) were included, of which 197 (73 %) participated in all four data collections. We reported further details on sample selection and data acquisition procedures in the Additional file 1. Informed consent was signed by the students prior to the participation to the study.

### Description of the educational context

Table 1 summarises the teaching and evaluation formats for each study year as well as the learning outcomes measures in this study. A full description is available in the Additional file 1. Briefly the pre-graduate medical curriculum has a duration of six years and is designed to provide a student-centred and integrated approach to students’ acquisition of theoretical knowledge and clinical competencies. The first study year is taught by lectures and assessed by a high-stake computer-based exam (CBE) constituted of multiple-choice-questions (MCQ) (factual knowledge). The second and third preclinical years are taught mainly by problem-based learning, assessed by a CBE and an oral examinations consisting of problem-solving questions (applied medical knowledge) in parallel with a clinical skills training, assessed by Objective-Structured-Clinical-Examination (OSCE). The fourth and fifth years are devoted to clinical training, through Problem-Solving activities and rotations in clinical clerkships, assessed by a CBE constituted of clinical vignettes and structured oral examination (applied clinical knowledge) and OSCEs (clinical skills). The sixth elective year finishes with the Swiss FLE comprising a high-stake written exam constituted of MCQs and a clinical skills part made of OSCEs.

**Table 1:** Description of the teaching and evaluation formats by study year

Study Year–Teaching unit	Main teaching format	Evaluation format	Measured learning outcomes
Year 1-Modules A/B	Integrated lectures	High-stakes CBE (MCQ)	Factual medical knowledge
Year 2-Modules 1/2	Small group PBL tutorials Clinical skills training	CBE (vignettes) Oral examination	Applied medical knowledge
Year 3-Modules 3/4	Small group PBL tutorials Clinical skills training	CBE (vignettes) Oral examination, OSCEs	Applied medical knowledge Clinical skills
Year 4-ICRU	Small group problem solving	CBE (clinical vignettes)	Applied clinical knowledge
Year 4-LCE-I	Rotations through clerkships	CBE (clinical vignettes) Oral examination OSCEs	Applied clinical knowledge Clinical skills
Year 5-LCE-I	Rotations through clerkships	CBE (clinical vignettes) Oral examination OSCEs	Applied clinical knowledge Clinical skills
Year 5-LCE-II	Rotations through clerkships	Oral examination	Applied clinical knowledge
Year 6-Elective year	Elective clerkships	High-stakes written exam (clinical vignettes) High-stakes OSCEs	Applied clinical knowledge Clinical skills

*Notes*: *ICRU* Introduction to clinical reasoning unit, *LCE-I* Learning in the clinical environment – first part, *LCE-II* Learning in the clinical environment – second part, *CBE* computer-based exam, *MCQ *multiple-choice questions, *PBL* problem-based learning, *OSCE* objective structured clinical examination

### Measures

#### Learning approaches

A French version of the Revised two-factor Study Process Questionnaire (R2-SPQ) [25] was created by translation and back-translation of two independent reviewers to measure students’ learning approaches across four assessments (i.e. Years 1, 2, 3 and 5) [26]. The R2-SPQ consists of 20 items scored on a 5-point Likert scale (from 1 = *this item is never or only rarely true of me* to 5 = *this item is always or almost always true of me*) with 10 items measuring DA and 10 items measuring SA. Examples of items tapping into DA and SA dimensions respectively are “I test myself on important topics until I understand them completely”, and “I find the best way to pass examinations is to try to remember answers to likely questions”. Total scores of DA and SA were calculated by summing up the scores of all the questions grouped under each dimension. Reliability results for DA (Cronbach’s *α* coefficients ranging from 0.74 to 0.84 across assessments) and SA (*α* coefficients ranging from 0.64 to 0.78) were aligned with previous studies [25, 27].

#### Learning outcomes

Learning outcomes were estimated from the exam scores for eight consecutive parts of the curriculum as described in Table 1. Exam scores were standardized so to have a mean of 0 and a standard deviation of 1, and were then summed and averaged by exam session to obtain repeated performance scores from pre-clinical throughout clinical training years [28]. Bivariate correlations between different exams within identical sessions ranged from 0.10 to 0.62 and were all significant at *p* < 0.01. Bivariate correlations between standardized exam scores across sessions ranged from 0.18 to 0.68 and were all significant at *p* < 0.01. These results support the analytical approach to combine scores from these different examinations into unique performance scores across academic years.

#### Covariates

Reading from previous research [29, 30], the following variables were adopted as covariates in the analyses: age (in years), gender (0 = *males*, 1 = *females*), and repeater^1^ status (0 = *non repeater*, 1 = *repeater*). We also controlled for differences according to enrolment year (1 = *2011*, 2 = *2012*, 3 = *2013*).

### Data analyses

An extended description of the analytical procedure adopted in the current study is presented in the Additional file 1. Preliminary analyses assessed accuracy of data entry, missing data and normality. Longitudinal analyses were divided into four steps. (1) First, latent growth modeling (LGM) [31], estimated the average initial level (henceforth intercept) and rate of change (slope) of DA and SA across assessments (from Year 2 to 5). (2) Second, to examine whether different trajectories of learning emerged from the total sample, group-based trajectory modeling (GBTM) [32] analysed the heterogeneity in the development of both DA and SA. GBTM is an exploratory approach assuming that the observed population is composed by a mixture of underlying trajectory subgroups. When testing GBTM models, different subgroup solutions are specified. The best-fitting model is then selected based on the goodness-of-fit indexes and theoretical considerations. (3) Third, after having identified different trajectories of DA and SA, Poisson regression with robust standard estimation method determined whether any covariate explained the likelihood of trajectory membership. (4) Finally, repeated measures analysis of covariance was conducted to assess the effect of time and trajectory membership on standardized exam scores across study years controlling for age, gender, enrolment year and repeater status. Stata 15 (StataCorp. 2015. Stata Statistical Software: Release 15. College Station, TX: StataCorp LP) was used for data analyses.

## Results

### Preliminary analyses

Table 2 reports descriptive statistics for all variables included in the analyses. The initial check of data for careless responding patterns across R2-SPQ’s items [33] revealed one case in Year 1 and two cases in Year 5 who were therefore coded as missing. Overall missing rates on learning approaches scores were 19.3 % in Year 1, 11.9 % in Year 2, 5.6 % in Year 3 and 11.2 % in Year 5. Little’s test was not significant (*χ*^2^ = 41.20, *df* = 40, *p* = 0.418), confirming that data were missing completely at random. Full information on maximum likelihood estimation method was thus used in the LGM and GBTM analyses. List-wise deletion method was adopted in the Poisson regression model and repeated measures analysis of covariance. This decision was further supported by the absence of multivariate outliers [34] and by absolute values of skewness and kurtosis below 1 and 6 respectively, thus suggesting that learning approaches and standardized exam scores were reasonably normally distributed [35].

**Table 2:** Descriptive statistics for all variables included in the analyses (*N* = 269). Standardized exam scores are reported

Variable	Range	Mean	SD
Age at Year 1	18–38	20.85	1.92
Female (*n* = 158, 59 %)			
Repeater (*n* = 141, 53 %)			
DA			
Year 1	17–50	33.46	5.69
Year 2	16–49	34.21	5.46
Year 3	17–46	32.62	6.11
Year 5	11–47	29.43	6.59
SA			
Year 1	10–37	22.24	4.78
Year 2	11–40	21.55	5.10
Year 3	11–41	23.65	5.84
Year 5	11–39	23.02	5.91
Exam sessions^a^			
Year 1-Modules A/B	0–100	62.17	12.74
Year 2-Modules 1/2	0–100	67.29	14.98
Year 3-Modules 3/4	0–100	50.93	17.94
Year 4-ICRU	0–100	60.54	17.70
Year 4-LCE-I	0–100	53.72	18.60
Year 5-LCE-I	0–100	61.98	16.13
Year 5-LCE-II	0–100	49.22	18.05
Year 6-Elective year	0–100	55.91	18.89

*Notes*. *DA* Deep learning approaches, *SA* Surface learning approaches, *ICRU* Introduction to clinical reasoning unit, *LCE-I* Learning in the clinical environment – first part, *LCE-II* Learning in the clinical environment – second part

^a^Standardized performance scores per exam session were expressed on a scale from 0 to 100 to ease interpretation

### Identifying trajectories of learning approaches

**Fig. 1:**
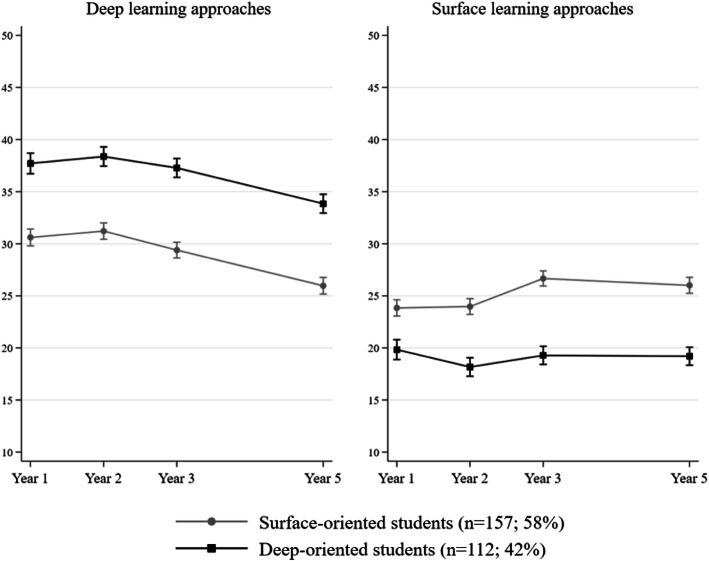
Expected scores in deep and surface learning approaches across assessments by trajectory groups with 95 % confidence intervals

Examination of fit indexes resulting from the LGM analysis (Table A) confirmed that DA declined (Slope mean = -1.10, SE = 0.23, *p* < 0.001) while SA increased across assessments (Slope mean = 0.66, SE = 0.22, *p* = 0.002). GBTM analyses indicated that the two-group solution was the best one according to differences in fit indexes between different solutions, interpretability and parsimonious considerations (Table B). The trajectories for both groups are illustrated in Fig. 1. Mean differences in learning approaches between trajectory groups were all significant at *p* < 0.001 at each assessment year. The *deep-oriented* group included students (*n* = 112; 42 %) predominantly using DA and little SA. The *surface-oriented* group included students (*n* = 157; 58 %) using more SA and less DA than their *deep-oriented* peers. According to post hoc analyses (Table C), *deep-oriented* students were fairly stable in their use of DA and SA but reported a significant decrease in DA between Year 3 and 5 and a small significant increase in SA between Years 2 and 3. Conversely, *surface-oriented* students reported a steady decline in DA from Year 2 to Year 5 and a significant increment in SA between Year 2 and 3, then stable between Years 3 and 5. Reading from Poisson regression analyses, trajectory subgroup membership was not explained by age, gender, repeater status or enrolment year.

### Learning outcomes by trajectory groups

**Fig. 2:**
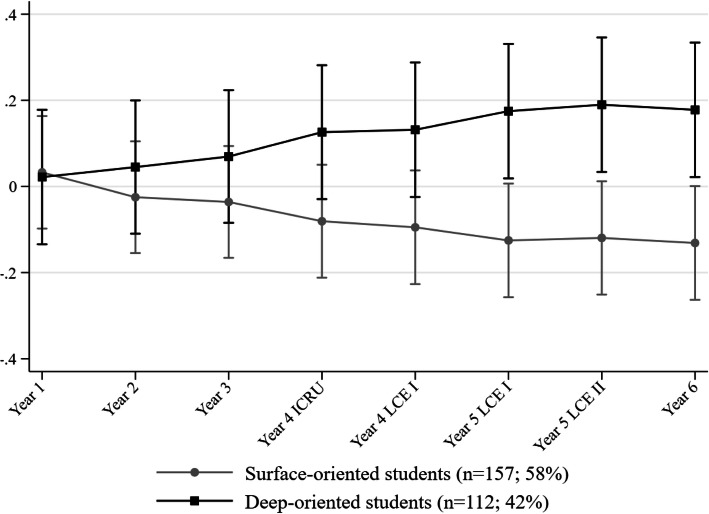
Expected standardized exam scores across years by trajectory groups with 95 % confidence intervals. *Notes*. ICRU: Introduction to clinical reasoning unit. LCE-I: Learning in the clinical environment – first part. LCE-II: Learning in the clinical environment – second part

Repeated measures analysis of covariance tested the effect of time and trajectory membership on standardized exam scores across study years while controlling for age, gender, enrolment year and repeater status. Trajectory membership had a significant direct effect on performance [*F*(1, 1760) = 6.88, *p* = 0.009] and a significant interaction effect with time [*F*(7, 1760) = 2.53, *p* = 0.014], meaning that learning outcomes differed across time in a different way according to trajectory groups. In order to ease the interpretation of these results, linear predictions for student standardized performance scores were graphically plotted for both groups across study years (Fig. 2). Examinations of contrasts between the two trajectory groups on standardized performance scores along study years (Table D) showed that *deep-oriented* students diverged toward better learning outcomes in comparison with their *surface-oriented* peers from Year 4 onwards. Unconditional of age, gender, enrolment year and repeater status, *deep-oriented* students were 21 % more likely to report higher performance scores than their *surface-oriented* peers. During Years 5 and 6, the odds for a better performance outcome were again in favour of *deep-oriented* students by more than 30 %. Further post hoc analyses by the means of independent sample t-tests with Bonferroni adjustments showed that performance scores were significantly different at *p* < 0.005 between trajectory sub-groups in Year 5 – LCE-II and Year 6 (Table D).

## Discussion

In this study, we modelized subgroups of medical students who evolve differently in their use of learning approaches along the curriculum, and – most importantly – who have different long-term clinical learning outcomes. To our best knowledge, evidencing these trajectories as well as their outcomes is a novel finding. In addition, whereas a lot of work has been done on other samples of university students, essentially concerning their academic performance, our results extend previous findings by showing that learning approaches also have an impact on the acquisition of clinical knowledge and skills. In our student sample, we found two groups with different trajectories of learning approaches. Students starting higher in SA and lower in DA at the onset of their studies tended to increase their use of SA across study years, while decreasing in DA. On the other hand, students starting higher in DA and lower in SA remained more stable on their use of DA and SA across study years, but still reported a slight decrease in DA during clinical years. We identified these two trajectory groups as s*urface-oriented* and *deep-oriented* students, respectively. Even if medical students are naturally oriented to use DA at the beginning of their studies [36], more than half of our sample tended to use SA and even slightly reinforced this tendency especially during pre-clinical training. Thus, like other studies adopting person-oriented analytical approaches in higher education research settings [21, 37, 38], we observed in this prospective long-term cohort of medical students, subgroups exhibiting different initial profiles in learning approaches (i.e. their spontaneous use of DA vs. SA at the onset of studies) and specific trajectories. Whereas Balasooriya et al. [22] described contradictory trajectories of learning approaches in subgroups of students after a single course unit, the trajectories observed in this study remained fairly parallel during the full medical curriculum.

Thus, if deep learning approaches are important predictors of academic performance, the essential question is how to promote students’ deep learning. An important amount of studies leads to contradictory results about the potential effect of the teaching context [23]. On the other side, several authors suggested that students could have a predisposition towards using either surface or deep learning, or that they might adapt or not their learning approaches depending on their personal profiles [18–21]. Therefore, evidence suggests that depending on various personal attributes, in particular their preferred learning approaches, students might not react the same to a given learning environment [13]. Our results are consistent with this hypothesis and confirm that students’ use of learning approaches at the onset of their studies is more predictive of the subsequent use of learning approaches than the educational context itself [19, 21]. Students constitute in fact a heterogeneous population whose individuals react and adapt differently in identical educational environments. This corroborates previous reports showing that even the most carefully designed learner-centered teaching environment might induce or not the use of DA [22]. An underlying reason for this heterogeneity might be connected to how students perceive their educational context [14, 39, 40]. It has been shown that *surface-oriented* medical students struggle to recognize the relevance of the educational context with regard to their future practice, have pre-existing beliefs that learning is linear, and feel confused with learner-centred methods [41]. On the other hand, the environment provided by medical studies may be stressful since it is characterized by a high workload, a competitive climate, complexity and uncertainty of knowledge, and high stakes assessment requirements. Taken together, these conditions could be experienced very differently among students, and create for the more fragile and less resilient of them, stress and anxiety pushing them towards using SA and impacting negatively their performance [42–44].

This study also demonstrates that *deep-oriented* students have better long-term clinical learning outcomes, thus confirming and extending at a longitudinal cohort level previous research [10, 11, 45]. These findings suggest that “one size does not fit all” and that medical educators should tailor the educational context to address this heterogeneous population of students. Nevertheless, several educational strategies might help *surface-oriented* students to reinforce the use of deep approaches. First, enhancing internal motivation [46] could potentially increase the initial use of DA [7, 29, 47]. Second, working on the beliefs and preconceptions that students have on learning may improve their perception of the educational context and foster the use of DA [41, 48]. Third, students’ social identity has been associated with how they perceive their academic environment and its relevance for their future practice, and is linked to an increased use of DA and a higher academic performance [49]. Finally, helping students to cope with stress will also promote use of DA [43, 44]. Identifying some of the factors that positively impact on the adoption of DA might contribute to define targeted strategies directed to students in need for guidance on their approach to learning. Furthermore, setting goals for reflective learning and writing with appropriate feedback have proved useful for promoting learners’ self-regulation strategies [50] and strengthening deep learning [51].

### Strengths, limitations and suggestions for future research

The main strength of this study is its person-oriented longitudinal design in which students have been followed during an entire curriculum and surveyed at different time points. Whereas the methodology in itself is not novel in the field of higher education research, to the best of our knowledge, no previous study has combined variable- and person-centered longitudinal approaches in the field of the medical education for research questions applied to learning approaches and academic performance. It certainly has limitations. First of all, these findings relate to a single institution and may not generalize to other contexts, even more since the number of students of this cohort is relatively modest. Second, although beyond the scope of the current study, several determinants of students’ learning profiles were not included in the current analyses although they may contribute to explain both initial levels and longitudinal changes in DA and SA. Future studies may thus extend the person-oriented model adopted here by investigating the individual characteristics that are inherent to a given trajectory. Moreover, as reminded in our analyses, such analytical approach is exploratory by definition [52]. More studies are thus needed to replicate and eventually validate the two trajectories of learning approaches observed here across different contexts and samples of medical students. Finally, future research may evaluate whether and how identify *surface-oriented* students as early as possible so to accompany and help them coping with their learning difficulties. In this perspective, making students aware of their predispositions towards surface learning could represent a first step towards changing. In addition, interventions to reinforce coping, motivation, beliefs and professional identity, are also able to promote DA. In our own practice as educators, we identified that favouring a reflective practice could be a way of addressing these issues.

## Conclusions

Despite the above limitations, this study contributes to the understanding of how medical students adopt learning approaches throughout their studies and how this impacts their learning outcomes. Our results suggest that students’ longitudinal trajectories of deep and surface learning approaches reflect their initial levels and that this is significantly associated with their clinical learning outcomes. Next steps should focus on testing the effectiveness of follow-up programs intended for medical students in need for guidance, to provide them with the best possible learning experience and equipping them to meet the challenges of the medical practice.

## Supplementary Information

**Additional file 1: Table A.** Comparison of fitted latent growth models for deep and surface learning approaches (*N* = 269). **Table B.** Group frequencies and fit indices based on estimated posterior probabilities for group-based trajectory modeling analysis of deep and surface learning approaches with different numbers of latent trajectory groups (*N* = 269). **Table C.** Comparisons of average scores in DA and SA between assessments and by trajectory groups based on paired sample t-tests. **Table D.** Differences in performance scores at each exam session between trajectory subgroups and contrasts between trajectory subgroups on standardized performance scores along exam sessions. Values are mean (standard deviations) unless otherwise stated (*N* = 269)

## Data Availability

The datasets generated and/or analysed during the current study are not publicly available due to the privacy of the students but are available from the corresponding author on reasonable request.

## Abbreviations

DA: Deep learning approaches
SA: Surface learning approaches
FLE: Federal licensing exam
CBE: Computer-based exam
MCQ: Multiple-choice-questions
OSCE: Objective-Structured-Clinical-Examination
R2-SPQ: Revised two-factor Study Process Questionnaire
LGM: Latent growth modeling
GBTM: Group-based trajectory modeling

## Footnotes

1 Students were classified as ‘repeaters’ if they repeated their pre-selection/first academic year.

## References

[1] Marton F, Säljö R (1976). On qualitative differences in learning: I—Outcome and process. Br J Educ Psychol.

[2] Biggs JB (1985). The role of metalearning in study process. Br J Educ Psychol.

[3] Salamonson Y, Weaver R, Chang S, Koch J, Bhathal R, Khoo C, Wilson I (2013). Learning approaches as predictors of academic performance in first year health and science students. Nurse Educ Today.

[4] Yonker JE (2011). The relationship of deep and surface study approaches on factual and applied test-bank multiple‐choice question performance. Assess Eval Higher Educ.

[5] Chamorro-Premuzic T, Furnham A (2008). Personality, intelligence and approaches to learning as predictors of academic performance. Pers Indiv Diff.

[6] Diseth Å (2007). Approaches to learning, course experience and examination grade among undergraduate psychology students: testing of mediator effects and construct validity. Stud High Educ.

[7] Everaert P, Opdecam E, Maussen S (2017). The relationship between motivation, learning approaches, academic performance and time spent. Acc Educ.

[8] Kassirer JP (2010). Teaching clinical reasoning: case-based and coached. Acad Med.

[9] Piumatti G, Abbiati M, Gerbase MW, Baroffio A (2021). Patterns of Change in Approaches to Learning and Their Impact on Academic Performance Among Medical Students: Longitudinal Analysis. Teach Learn Med.

[10] May W, Chung E-K, Elliott D, Fisher D (2012). The relationship between medical students’ learning approaches and performance on a summative high-stakes clinical performance examination. Med Teach.

[11] Feeley AM, Biggerstaff DL (2015). Exam Success at Undergraduate and Graduate-Entry Medical Schools: Is Learning Style or Learning Approach More Important? A Critical Review Exploring Links Between Academic Success, Learning Styles, and Learning Approaches Among School-Leaver Entry (“Traditional”) and Graduate-Entry (“Nontraditional”) Medical Students. Teach Learn Med.

[12] McManus I, Keeling A, Paice E (2004). Stress, burnout and doctors’ attitudes to work are determined by personality and learning style: A twelve year longitudinal study of UK medical graduates. BMC Med.

[13] Price L. Modelling factors for predicting student learning outcomes in higher education. In D. Gijbels, V. Donche, J. T. E. Richardson, and J. D. Vermunt (Eds.). Learning patterns in higher education: Dimensions and research perspectives. London: Routledge; 2014. p. 56–77.

[14] Baeten M, Kyndt E, Struyven K, Dochy F (2010). Using student-centred learning environments to stimulate deep approaches to learning: Factors encouraging or discouraging their effectiveness. Educ Res Rev.

[15] Struyven K, Dochy F, Janssens S, Gielen S (2006). On the dynamics of students’ approaches to learning: the effects of the teaching/learning environment. Learn Instruct..

[16] Gijbels D, Segers M, Struyf E (2008). Constructivist learning environments and the (im)possibility to change students’ perceptions of assessment demands and approaches to learning. Instr Sci.

[17] Asikainen H, Gijbels D (2017). Do students develop towards more deep approaches to learning during studies? A systematic review on the development of students’ deep and surface approaches to learning in higher education. Educ Psychol Rev.

[18] Biggs JB (1993). The process of learning / John B. Biggs, Phillip J. Moore.

[19] Coertjens L, Vanthournout G, Lindblom-Ylänne S, Postareff L (2016). Understanding individual differences in approaches to learning across courses: A mixed method approach. Learning Individual Differences.

[20] Fryer LK, Vermunt JD (2018). Regulating approaches to learning: Testing learning strategy convergences across a year at university. Br J Educ Psychol.

[21] Kyndt E, Dochy F, Struyven K, Cascallar E (2012). Looking at learning approaches from the angle of student profiles. Educational Psychology.

[22] Balasooriya CD, Toohey S, Hughes C (2009). The cross-over phenomenon: unexpected patterns of change in students’ approaches to learning. Stud High Educ.

[23] Baeten M, Struyven K, Dochy F (2013). Student-centred teaching methods: Can they optimise students’ approaches to learning in professional higher education?. Studies in Educational Evaluation.

[24] Muthén B, Muthén LK (2000). Integrating person-centered and variable‐centered analyses: Growth mixture modeling with latent trajectory classes. Alcoholism: Clinical Experimental Research.

[25] Biggs JB, Kember D, Leung DYP (2001). The revised two factor study process questionnaire: R-SPQ-2F. British J Educational Psychology.

[26] Abbiati M, Baroffio A, Gerbase MW (2016). Personal profile of medical students selected through a knowledge-based exam only: are we missing suitable students?. Medical education online.

[27] Justicia F, Pichardo MC, Cano F, Berbén ABG, De la Fuente J (2008). The revised two-factor study process questionnaire (R-SPQ-2F): Exploratory and confirmatory factor analyses at item level. Eur J Psychol Educ.

[28] Griffin B, Bayl-Smith P, Hu W (2018). Predicting patterns of change and stability in student performance across a medical degree. Medical education.

[29] Piumatti G, Abbiati M, Baroffio A, Gerbase MW (2018). Associations between motivational factors for studying medicine, learning approaches and empathy among medical school candidates. Adv Health Sci Educ.

[30] Zhou Y-X, Zhao Z-T, Li L, Wan C-S, Peng C-H, Yang J, Ou C-Q (2014). Predictors of first-year GPA of medical students: a longitudinal study of 1285 matriculates in China. BMC Med Educ.

[31] Duncan TE, Duncan SC, Strycker LA. An introduction to latent variable growth curve modeling: concepts, issues, and application. New York: Routledge. 2013.

[32] Nagin D. Group-based modeling of development. Cambridge: Harvard University Press.

[33] Meade AW, Craig SB (2012). Identifying careless responses in survey data. Psychol Methods.

[34] Billor N, Hadi AS, Velleman PF (2000). BACON: blocked adaptive computationally efficient outlier nominators. Comput Stat Data Anal.

[35] Kline RB (2015). Principles and practice of structural equation modeling.

[36] Chonkar SP, Ha TC, Chu SSH, Ng AX, Lim MLS, Ee TX, Ng MJ, Tan KH (2018). The predominant learning approaches of medical students. BMC Med Educ.

[37] Bouckenooghe D, Cools E, De Clercq D, Vanderheyden K, Fatima T (2016). Exploring the impact of cognitive style profiles on different learning approaches: empirical evidence for adopting a person-centered perspective. Learn Individ Diff.

[38] Vanthournout G, Coertjens L, Gijbels D, Donche V, Van Petegem P (2013). Assessing students’ development in learning approaches according to initial learning profiles: A person-oriented perspective. Stud Educ Eval.

[39] Karagiannopoulou E, Milienos FS (2015). Testing two path models to explore relationships between students’ experiences of the teaching–learning environment, approaches to learning and academic achievement. Educ Psychol.

[40] Gustin M-P, Abbiati M, Bonvin R, Gerbase MW, Baroffio A (2018). Integrated problem-based learning versus lectures: a path analysis modelling of the relationships between educational context and learning approaches. Med Educ Online.

[41] Balasooriya CD, Tetik C, Harris P (2011). Why is my design not working? The role of student factors. Res Papers Educ.

[42] LeBlanc VR (2009). The effects of acute stress on performance: implications for health professions education. Acad Med.

[43] Behzadnia A, Smith D, Goodson M (2018). A cross-sectional examination of the relationship between approaches to learning and perceived stress among medical students in Malaysia. Educ Health.

[44] Sandover S, Jonas-Dwyer D, Marr T (2015). Graduate entry and undergraduate medical students’ study approaches, stress levels and ways of coping: a five year longitudinal study. BMC Med Educ.

[45] Liang J-C, Chen Y-Y, Hsu H-Y, Chu T-S, Tsai C-C (2018). The relationships between the medical learners’ motivations and strategies to learning medicine and learning outcomes. Med Educ Online.

[46] Pelaccia T, Viau R (2017). Motivation in medical education. Med Teach.

[47] Kyndt E, Dochy F, Struyven K, Cascallar E (2011). The direct and indirect effect of motivation for learning on students’ approaches to learning through the perceptions of workload and task complexity. Higher Educ Res Dev.

[48] Dart BC, Burnett PC, Purdie N, Boulton-Lewis G, Campbell J, Smith D (2000). Students’ Conceptions of Learning, the Classroom Environment, and Approaches to Learning. J Educ Res.

[49] Bliuc A-M, Ellis RA, Goodyear P, Hendres DM (2011). The role of social identification as university student in learning: relationships between students’ social identity, approaches to learning, and academic achievement. Educ Psychol.

[50] Uygur J, Stuart E, De Paor M, Wallace E, Duffy S, O’Shea M, Smith S, Pawlikowska T (2019). A Best Evidence in Medical Education systematic review to determine the most effective teaching methods that develop reflection in medical students: BEME Guide No. 51. Med Teach.

[51] Tsingos C, Bosnic-Anticevich S, Smith L (2015). Learning styles and approaches: can reflective strategies encourage deep learning?. Curr Pharm Teach Learn.

[52] Piumatti G, Abbiati M, Baroffio A, Gerbase MW. Empathy trajectories throughout medical school: relationships with personality and motives for studying medicine. Adv Health Sci Educ. 2020;25(5):1227–42. 10.1007/s10459-020-09965-y 32095990

