# Supplementary material for: Trajectories of learning approaches during a full medical curriculum: impact on clinical learning outcomes
Source: BMC Med Educ. 2021 Jul 7;21:370. doi: 10.1186/s12909-021-02809-2 (PMC8262035; doi:10.1186/s12909-021-02809-2)
Supplement: Supplementary file 1 — Additional file 1: Table A. Comparison of fitted latent growth models for deep and surface learning approaches (N = 269). Table B. Group frequencies and fit indices based on estimated posterior probabilities for group-based trajectory modeling analysis of deep and surface learning approaches with different numbers of latent trajectory groups (N = 269). Table C. Comparisons of average scores in DA and SA between assessments and by trajectory groups based on paired sample t-tests. Table D. Differences in performance scores at each exam session between trajectory subgroups and contrasts between trajectory subgroups on standardized performance scores along exam sessions. Values are mean (standard deviations) unless otherwise stated (N = 269) [file 12909_2021_2809_MOESM1_ESM.docx]

**Trajectories of learning approaches during a full medical curriculum:**

**Impact on clinical learning outcomes**

**Appendix**

This section provides the details of (1) the educational context, and (2) the sample selection and analyses using latent growth modeling (LGM) and group-based trajectory modeling (GBTM) in Stata 15 (StataCorp. 2015. Stata Statistical Software: Release 15. College Station, TX: StataCorp LP).

**Description of the educational context**

The pre-graduate medical curriculum has a duration of six years and is designed to provide a student-centered and integrated approach to students’ acquisition of theoretical knowledge and clinical competencies. The first pre-selection study year is organised as two multidisciplinary integrated modules taught by lectures and assessed by a high-stake computer-based exam (CBE) constituted of multiple-choice-questions (MCQ) (pass rate 42%). During the second and third preclinical years, all students take part in four sequential modules consisting of multidisciplinary thematic teaching units. The units are taught mainly by problem-based learning in small groups combined with a few seminars, integrated lectures, practical sessions and clinical skills training. Knowledge and skills acquired during every teaching unit are assessed at the end of each module by a CBE referring to multidisciplinary vignettes and by practical oral examinations, and clinical skills by a single Objective-Structured-Clinical-Examination (OSCE) exam at the end of the third year (success rate 90%). At the beginning of the fourth year, students learn the basics of clinical reasoning in an Introduction to Clinical Reasoning Unit (ICRU) through problems of various clinical disciplines learnt in small groups by Problem-Solving derived from PBL. During the second part of the fourth year and the fifth year, students are assigned to seven different rotations of 10 clinical clerkships and 3 transversal disciplines (Learning in the Clinical Environment LCE-I and II). Learning activities are integrated during these clinical rotations and consist of case-based clinical reasoning tutorials and various interactive seminars two days a week. The rest of the week, students act as interns in the healthcare team of a given clinical unit. Clinical reasoning skills, professionalism and clinical interactions are formatively evaluated while working under direct supervision, and summatively at the end of each semester using CBE constituted of clinical vignettes, structured oral examination and OSCEs (success rate 90-95%). The last elective year finishes with a high-stakes licensing exam certifying the pre-graduate medical training. The Swiss federal licensing examination (FLE) comprises a high-stake written exam constituted of 300 MCQs and a clinical skills (CS) part made of 12 station OSCE. The national examination committee defines the pass score for the MCQ on the basis of two content-related methods, for the CS on the basis of borderline regression method [1]. Candidates have to meet pass scores in both parts of the examination in order to overall pass the FLE. Success rates average around 96.6% for the MCQ and 98.1% for the CS exam.

1. **Sample selection and analyses**

In 2011, 340 students initially took part to the study, 92 (28%) of which were

repeating their pre-selection academic year for the second time after having failed to be

admitted to the 2nd academic year. In 2012, 349 students participated, 86 (25%) of which

were repeaters that already participated to the study the year before and 8 (2%) were repeaters participating to the study for their first time. In order not to lose those students that did participate in 2012 but failed to be admitted to the 2nd academic year, in 2013 we recruited a group of 45 repeaters enrolled for their second time to the pre-selection academic year, 39 (87%) of which already participated to the study the precedent year of data collection and 6 (13%) participating to the study for their first time. In sum, based on the selection criteria for our longitudinal analyses (i.e. participating to at least three data collection assessments across taking place during the 1st, 2nd, 3rd and 5th academic years) we retained 90 students with baseline measures collected in 2011, 144 in 2012 and 34 in 2013.

To determine the shape of the trajectories for deep and surface learning we followed the procedure suggested by Phan [2], by testing three alternative models: (1) a no-growth model where no slope component was assumed; (2) a linear growth model assuming a linear pattern of change across assessments by fixing slope parameters to 0 at Year 1, to 1 at Year 2, to 2 at Year 3 and to 4 at Year 5; and (3) a nonlinear growth model where the form of change across assessments is not specified a priori and the slope parameter is fixed to 0 at Year 1 and to 4 at Year 5 to allow a separation of the intercept and slope components at baseline assessment and provide a scale of measurement for the slope. In this last model the slope parameters at Years 2 and 3 were freely estimated. To assess model comparison between each solution, we used the *χ*^2^ difference test. In addition, overall model fit was evaluated using the following fit indexes: the Bentler comparative fit index (CFI) [3], the Tucker Lewis index (TLI) [4], and the Steiger-Lind root mean square error of approximation (RMSEA) [5]. Models with CFI and TLI values over 0.90 and RMSEA values below 0.05 are considered an acceptable fit. Since the RMSEA often falsely indicated a poor fitting model when having small degree of freedoms [6], we adopted different indexes at the same time to provide a more reliable assessment of model fit. Comparison between different LGM models are reported in **Table A**.

After determining the trajectories of deep and surface learning by estimating mean and variance of intercept (initial level) and slope (rate of change) latent measures, analyses were extended into GBTM. More specifically, the LGM analytical step tested the hypothesis of significant variance in the initial level and rate of individual change in learning approaches across assessments. Subsequently, GBTM tested the hypothesis that there were groups of students within the data that follow distinctive longitudinal trajectories [7]. GBTM in Stata 15 uses the maximum likelihood estimation method by a general quasi-Newton procedure [8] to predict the trajectory of each group and calculate the probability of membership in a specific group for each participants [9]. GBTM is an explorative approach since the number of trajectory groups are not known a priori. To determine the best solution, we looked at Akaike’s information criterion (AIC) [10], Bayesian information criterion (BIC) and adjusted Bayesian information criterion (aBIC) [11] across alternative models, with smaller absolute values indicating a better fit to the data [7]. As there are no definitive decision criteria for the optimal number of groups, the selection of the best solution was further carried out on the basis of parsimony and interpretability of the results [12].

Based on LGM, intercept’s variance was significant at *p* < 0.05 for both measures of learning approaches, while slope’s variance was significant at *p* < 0.05 only for DA, suggesting heterogeneity in both initial levels and rate of change for DA and in initial levels for SA across academic years. Analyses were thus extended into GBTM to identify trajectories of learning approaches. As reported in **Table B**, up to the 4-group solution all statistical indexes diminished indicating better fit to the data. The 5-group solution listed a group with a very low percentage of participants (i.e. 3%) and was therefore considered not acceptable. Reading from LGM’s results, we estimated each group solution adopting a non-linear curve that would best resemble deep and surface learning trajectories. Models with 3 and 4 groups were further graphically investigated as BIC, aBIC and AIC values began to level out at the 2-group solution. The 3- and 4-group solutions added no additional information that was not previously represented by the 2-group solution. In particular, solutions with more than 2 groups appeared to simply split these initial groups into smaller classes that did not help to better describe the data. Average group membership probability for the 2-group solution was equal to 0.95 for the first group and 0.94 for the second group indicating a high degree of accuracy. Therefore, a 2-group model was retained for further analyses.

References

1. Guttormsen S, Beyeler C, Bonvin R, Feller S, Schirlo C, Schnabel K, Schurter T, Berendonk C: **The new licencing examination for human medicine: from concept to implementation**. *Swiss Med Wkly* 2013, **143**:w13897.

2. Phan HP: **Deep processing strategies and critical thinking: Developmental trajectories using latent growth analyses**. *The Journal of Educational Research* 2011, **104**(4):283-294.

3. Bentler PM: **Comparative fit indexes in structural models**. *Psychological Bulletin* 1990, **107**(2):238-246.

4. Bentler PM, Bonett DG: **Significance tests and goodness of fit in the analysis of covariance structures**. *Psychological Bulletin* 1980, **88**(3):588-606.

5. Steiger JH: **Structural model evaluation and modification: An interval estimation approach**. *Multivariate Behavioral Research* 1990, **25**(2):173-180.

6. Kenny DA, Kaniskan B, McCoach DB: **The performance of RMSEA in models with small degrees of freedom**. *Sociological Methods & Research* 2015, **44**(3):486-507.

7. Nagin DS: **Analyzing developmental trajectories: a semiparametric, group-based approach**. *Psychological Methods* 1999, **4**(2):139-157.

8. Dennis Jr JE, Gay DM, Walsh RE: **An adaptive nonlinear least-squares algorithm**. *ACM Transactions on Mathematical Software (TOMS)* 1981, **7**(3):348-368.

9. Jones BL, Nagin DS: **A Stata plugin for estimating group-based trajectory models**. *Research Showcase@ CMU Carnegie Mellon University Retrieved on July* 2012, **10**:2015.

10. Akaike H: **A new look at the statistical model identification**. *IEEE transactions on automatic control* 1974, **19**(6):716-723.

11. Raftery AE: **Bayesian model selection in social research**. *Sociological Methodology* 1995, **25**:111-163.

12. Collins LM, Lanza ST: **Latent class and latent transition analysis: With applications in the social, behavioral, and health sciences**, vol. 718: John Wiley & Sons; 2013.

**Table A.** Comparison of fitted latent growth models for deep and surface learning approaches (*N* = 269)

| **Model** | ***χ*^2^** | ***df*** | ***p*** | **CFI** | **TLI** | **RMSEA** | **∆*χ*^2^** | **∆*df*** | ***p*(d)** |
| --- | --- | --- | --- | --- | --- | --- | --- | --- | --- |
| Deep approaches to learning |  |  |  |  |  |  |  |  |  |
| No growth | 181.70 | 8 | <0.001 | 0.560 | 0.670 | 0.284 |  |  |  |
| Linear growth | 43.57 | 5 | <0.001 | 0.902 | 0.883 | 0.169 | 138.13 | 3 | <0.001 |
| **Nonlinear growth** | **11.05** | **3** | **0.012** | **0.980** | **0.959** | **0.100** | **32.52** | **2** | **<0.001** |
| Surface approaches to learning |  |  |  |  |  |  |  |  |  |
| No growth | 76.24 | 8 | <0.001 | 0.794 | 0.845 | 0.178 |  |  |  |
| Linear growth | 41.92 | 5 | <0.001 | 0.888 | 0.866 | 0.166 | 34.32 | 3 | <0.001 |
| **Nonlinear growth** | **17.90** | **3** | **0.001** | **0.955** | **0.910** | **0.136** | **24.02** | **2** | **<0.001** |

*Notes*. CFI = Comparative Fit Index; TLI = Tucker–Lewis Index; RMSEA = root mean square error of approximation; ∆*χ*^2^ = difference in likelihood ratio tests; ∆*df* = difference in *df*; *p*(d) = probability of the difference texts. The best model solutions are marked in bold.

**Table B.** Group frequencies and fit indices based on estimated posterior probabilities for group-based trajectory modeling analysis of deep and surface learning approaches with different numbers of latent trajectory groups (*N* = 269)

| **Number of groups** | **BIC** | **aBIC** | **AIC** |
| --- | --- | --- | --- |
| 1 | -6018.15 | -6010.35 | -5995.97 |
| **2 (*n*_1_ = 58%; *n*_2_ = 42%)** | **-5803.17** | **-5788.53** | **-5761.57** |
| 3 (*n*_1_ = 46%; *n*_2_ = 40%; *n*_3_ = 14%) | -5784.81 | -5763.35 | -5723.80 |
| 4 (*n*_1_ = 41%; *n*_2_ = 25%; *n*_3_ = 22%; *n*_4_ = 12%) | -5762.85 | -5734.55 | -5682.43 |
| 5 (*n*_1_ = 29%; *n*_2_ = 20%; *n*_3_ = 24%; *n*_4_ = 24%; *n*_5_ = 3%) | -5764.58 | -5729.45 | -5664.75 |

*Notes*. BIC = Bayesian Information Criteria; aBIC = Adjusted Bayesian Information Criteria; AIC = Akaike Information Criteria. The chosen option is marked in bold.

**Table C.** Comparisons of average scores in DA and SA between assessments and by trajectory groups based on paired sample t-tests

|  | | **Year** | **M (SD)** | **Year 1** | | **Year 2** | | **Year 3** | |
| --- | --- | --- | --- | --- | --- | --- | --- | --- | --- |
|  |  |  |  | **Change (*p*)** | ***d*** | **Change (*p*)** | ***d*** | **Change (*p*)** | ***d*** |
| Surface-oriented learners  (*n* = 157) | Deep learning approaches | Year 1 | 30.61 (4.55) |  |  |  |  |  |  |
|  |  | Year 2 | 31.22 (4.46) | + (0.632) | 0.04 |  |  |  |  |
|  |  | Year 3 | 29.39 (4.89) | – (0.010) | 0.24 | **– (<0.001)** | **0.39** |  |  |
|  |  | Year 5 | 25.97 (5.52) | **– (<0.001)** | **0.78** | **– (<0.001)** | **1.01** | **– (<0.001)** | **0.67** |
|  | Surface learning approaches | Year 1 | 23.84 (4.30) |  |  |  |  |  |  |
|  |  | Year 2 | 23.97 (4.60) | – (0.840) | 0.02 |  |  |  |  |
|  |  | Year 3 | 26.67 (4.73) | **+ (<0.001)** | **0.46** | **+ (<0.001)** | **0.58** |  |  |
|  |  | Year 5 | 26.01 (5.38) | **+ (0.001)** | **0.31** | **+ (<0.001)** | **0.40** | **–** (0.140) | 0.13 |
| Deep-oriented learners  (*n* = 112) | Deep learning approaches | Year 1 | 37.71 (4.44) |  |  |  |  |  |  |
|  |  | Year 2 | 38.37 (3.72) | + (0.141) | 0.17 |  |  |  |  |
|  |  | Year 3 | 37.28 (4.46) | – (0.695) | 0.04 | – (0.018) | 0.25 |  |  |
|  |  | Year 5 | 33.85 (5.03) | **– (<0.001)** | **0.60** | **– (<0.001)** | **0.80** | **– (<0.001)** | **0.68** |
|  | Surface learning approaches | Year 1 | 19.84 (4.47) |  |  |  |  |  |  |
|  |  | Year 2 | 18.17 (3.63) | – (0.004) | 0.35 |  |  |  |  |
|  |  | Year 3 | 19.29 (4.35) | **–** (0.164) | 0.15 | **+ (0.002)** | **0.34** |  |  |
|  |  | Year 5 | 19.21 (4.08) | **–** (0.307) | 0.11 | + (0.024) | 0.24 | **–** (0.984) | 0.00 |

*Notes*. +: increase between consecutive years; –: decrease between consecutive years. Effect sizes (*d*) are based on Cohen’s classification: 0.2 = ‘small’, 0.5 = ‘medium’ and 0.8 = ‘large’. Significant results according to post hoc Bonferroni adjustments are marked in bold.

**Table D.** Differences in performance scores at each exam session between trajectory subgroups and contrasts between trajectory subgroups on standardized performance scores along exam sessions. Values are mean (standard deviations) unless otherwise stated (*N* = 269)

| Exam session | Surface-oriented learners ^a^  (*n* = 157) | Deep-oriented learners ^a^  (*n* = 112) | *p* ^b^ | Standardized performance score | | |
| --- | --- | --- | --- | --- | --- | --- |
|  |  |  |  | Contrast (SE) | *p* | CI_95_ |
| Year 1-Modules A/B | 62.32 (12.37) | 61.97 (13.30) | 0.831 | -0.01 (0.10) | 0.916 | -0.21, 0.19 |
| Year 2-Modules 1/2 | 66.68 (14.38) | 68.14 (15.80) | 0.437 | 0.07 (0.10) | 0.497 | -0.13, 0.27 |
| Year 3-Modules 3/4 | 49.99 (16.35) | 52.23 (19.95) | 0.320 | 0.11 (0.10) | 0.305 | -0.10, 0.31 |
| Year 4-ICRU | 59.15 (16.90) | 62.47 (18.67) | 0.137 | 0.21 (0.10) | 0.046 | 0.01, 0.41 |
| Year 4-LCE-I | 52.17 (17.35) | 55.87 (20.09) | 0.117 | 0.23 (0.10) | 0.030 | 0.02, 0.43 |
| Year 5-LCE-I | 60.15 (15.41) | 64.50 (16.83) | 0.033 | 0.30 (0.10) | 0.004 | 0.10, 0.50 |
| Year 5-LCE-II | **45.54 (16.88)** | **54.35 (18.05)** | **0.001** | 0.31 (0.10) | 0.003 | 0.11, 0.51 |
| Year 6-Elective year | **53.04 (18.92)** | **59.87 (18.20)** | **0.004** | 0.31 (0.10) | 0.003 | 0.11, 0.51 |

*Notes*. SE: Standard error. CI_95_: 95% Confidence intervals. ICRU: Introduction to clinical reasoning unit. LCE-I: Learning in the clinical environment – first part. LCE-II: Learning in the clinical environment – second part. Significant results according to post hoc Bonferroni adjustments are marked in bold.

^a^ Standardized performance scores per exam session were expressed on a scale from 0 to 100 to ease interpretation.

^b^ Statistical differences are based on independent samples t-tests.
